# Association between meniscal tears and the peak external knee adduction moment and foot rotation during level walking in postmenopausal women without knee osteoarthritis: a cross-sectional study

**DOI:** 10.1186/ar2428

**Published:** 2008-05-20

**Authors:** Miranda L Davies-Tuck, Anita E Wluka, Andrew J Teichtahl, Johanne Martel-Pelletier, Jean-Pierre Pelletier, Graeme Jones, Changhai Ding, Susan R Davis, Flavia M Cicuttini

**Affiliations:** 1 Department of Epidemiology and Preventive Medicine, Monash University, Central and Eastern Clinical School, 89 Commercial Road, Alfred Hospital, Melbourne, Victoria 3004, Australia; 2 Baker Heart Research Institute, 75 Commercial Road, Melbourne, Victoria 3004, Australia; 3 Osteoarthritis Research Unit, University of Montreal Hospital Centre, Notre-Dame Hospital, 1560 Sherbrooke Street East, Montreal, Quebec, H2L 4M1, Canada; 4 Menzies Research Institute, University of Tasmania, Level 2, Surrey House, 199 Macquarie Street, Hobart, Tasmania 7000, Australia; 5 National Health and Medical Research Council of Australia Centre of Clinical Research Excellence for the Study of Women's Health, Monash University Medical School, Alfred Hospital, Prahran, Victoria 3181, Australia

## Abstract

**Introduction:**

Meniscal injury is a risk factor for the development and progression of knee osteoarthritis, yet little is known about risk factors for meniscal pathology. Joint loading mediated via gait parameters may be associated with meniscal tears, and determining whether such an association exists was the aim of this study.

**Methods:**

Three-dimensional Vicon gait analyses were performed on the dominant knee of 20 non-osteoarthritic women, and the peak external knee adduction moment during early and late stance was determined. The degree of foot rotation was also examined when the knee adductor moment peaked during early and late stance. Magnetic resonance imaging was used to determine the presence and severity of meniscal lesions in the dominant knee.

**Results:**

The presence (*P *= 0.04) and severity (*P *= 0.01) of medial meniscal tears were positively associated with the peak external knee adduction moment during early stance while a trend for late stance was observed (*P *= 0.07). They were also associated with increasing degrees of internal foot rotation during late stance, independent of the magnitude of the peak external knee adduction moment occurring at that time (*P *= 0.03). During level walking among healthy women, the presence and severity of medial meniscal tears were positively associated with the peak external knee adduction moment. Moreover, the magnitude of internal foot rotation was associated with the presence and severity of medial meniscal lesions, independent of the peak knee adductor moment during late stance.

**Conclusion:**

These data may suggest that gait parameters may be associated with meniscal damage, although longitudinal studies will be required to clarify whether gait abnormalities predate meniscal lesions, or *vice versa*, and therefore whether modification of gait patterns may be helpful.

## Introduction

Meniscal injury is recognised as a significant risk factor for the development and progression of knee osteoarthritis (OA) [1,2] and may be present with or without a history of significant trauma when assessed via magnetic resonance imaging (MRI) [2-6]. In subjects without clinical knee OA, meniscal tears have been associated with structural changes associated with OA, including the presence of more severe cartilage defects, diminished tibial cartilage volume, and increased tibial bone area [7]. Therefore, determining which modifiable variables are associated with meniscal lesions, even among people with no clinical knee OA, may help to better understand the pathogenesis of knee OA and develop preventative strategies.

Recently, there has been increasing interest in the peak external knee adduction moment in epidemiological studies examining knee joint morphology and the genesis of knee OA and pain [8-11]. The peak external knee adduction moment, which is generated by the combination of the ground reaction force passing medial to the centre of the knee joint during gait and the perpendicular distance of this force from the centre of the knee joint, is a major determinant of 70% of the total knee joint load passing through the medial tibiofemoral compartment during walking [10]. Recently, we demonstrated that the degree of external foot rotation was associated with a reduction in the magnitude of the external peak knee adduction moment during healthy human walking [12]. This result was similar to the previous finding that a toe-out posture of the lower limb also reduced the magnitude of the peak knee adductor moment during late stance [13,14]. Given that the peak external knee adduction moment is a major determinant of the axial load passing through the medial tibiofemoral compartment and that the degree of foot rotation may help to mediate changes in this load, it is possible that these variables may also be associated with the presence of compartment-specific meniscal lesions. The aim of this cross-sectional study was to determine whether the peak external knee adduction moment and the degree of foot rotation occurring during level walking are associated with the presence and severity of meniscal lesions among women with no clinical knee OA.

## Materials and methods

### Subjects

Twenty women involved in an existing study of healthy aging [8] were recruited through a women's health clinic and advertising in the local media. The study was approved by the ethics committees of Alfred Hospital (Prahran, Victoria, Australia), Caulfield Hospital (Caulfield, Victoria, Australia), and La Trobe University (Melbourne, Victoria, Australia). All participants gave informed consent.

Exclusion criteria were a history of knee OA, radiological OA or any history of symptoms requiring medical treatment, any knee pain for more than 1 day in the month prior to testing, previous or planned knee joint replacement, inflammatory arthritis, malignancy, fracture in the last 10 years, contraindication to MRI (for example, pacemaker, cerebral aneurysm clip, cochlear implant, presence of shrapnel in strategic locations, metal in the eye, and claustrophobia), inability to walk 50 feet without the use of assistive devices, hemiparesis, and any other musculoskeletal, cardiovascular, or neurological condition that would impair normal gait as previously described [8].

### Data collection

Weight was measured to the nearest 0.1 kg (shoes, socks, and bulky clothing removed) using a single pair of electronic scales. Height was measured to the nearest 0.1 cm (shoes and socks removed) using a stadiometer. Body mass index (BMI) (weight in kilograms divided by height squared in metres squared) was calculated. A history of knee trauma and knee surgery was obtained.

### Magnetic resonance imaging

MRI was performed on the dominant knee (that is, the leg from which a subject stepped off from when initiating walking) as previously described [15]. The following sequence and parameters were used: a T1-weighted fat-suppressed three-dimensional gradient recall acquisition in the steady state; flip angle 55°; repetition time 58 ms; echo time 12 ms; field of view 16 cm; 60 partitions; 512 (frequency direction, superior-inferior) × 512 (phase-encoding direction, anterior-posterior) matrix; one acquisition, time 11 minutes 56 seconds. Sagittal images were obtained at a partition thickness of 1.5 mm and an in-plane resolution of 0.31 × 0.31 mm (512 × 512 pixels).

Meniscal tears were assessed in the sagittal view and confirmed in coronal and axial views by experienced radiologists (André Pelletier and Josée Thériault) as previously described [3,7,16]. The presence of a tear was based on the presence of a signal, which was line-shaped, brighter than the dark meniscus, and reached the surface of the meniscus at both ends within six defined regions (anterior horn, body and posterior horn at both medial and lateral tibiofemoral compartments). A semi-quantitative lesion assessment of meniscal tears was also performed. Our scoring system for meniscal damage referred to the accepted MRI nomenclature for meniscal anatomy, which is in accordance with arthroscopic literature [17]. The proportion of the menisci affected by tears was scored separately using the following semi-quantitative scale [3]: 0 = no damage; 1 = one out of three meniscal areas involved (anterior, middle, posterior horns); 2 = two out of three areas involved; 3 = all three areas involved. The intra- and inter-reader correlation coefficients ranged from 0.86 to 0.96 for the meniscal tears [16].

### Gait analysis

Gait analyses were conducted in the gait laboratory in the Musculoskeletal Research Centre, La Trobe University. A six-camera Vicon motion analysis system (Oxford Metrics Ltd., Oxford, UK) was used to capture three-dimensional kinematic data during four walking trials on the dominant leg at the subjects' self-selected speed to capture normal gait patterns. Ground reaction forces were measured by a Kistler 9281 force-platform (Kistler Instruments, Winterthur, Switzerland). Inverse dynamic analyses were performed using 'PlugInGait' (Oxford Metrics Ltd.), which is based on a previously proposed model [18], to obtain joint moments calculated about an orthogonal axis system located in the distal segment of a joint as previously described [8,12]. Inter-ASIS (anterior superior iliac spine) distance was measured using a calliper, allowing the medial-lateral and proximal-distal coordinates of the hip joint centre to be determined by the method previously described [18]. The ASIS to greater-trochanter measurement provided the anterior-posterior coordinate of the hip joint. A knee alignment device (KAD) was used to calculate knee joint axes. The coronal plane of the thigh was defined as the plane containing the hip joint centre, knee marker, and lateral KAD marker. The coronal plane of the shank contained the knee joint centre and lateral malleolus marker. The angle formed by the knee and ankle joint axes measured tibial torsion.

Foot rotation was measured about an axis perpendicular to the foot vector and the ankle flexion axis. It is defined as the angle between the foot vector and the sagittal axis of the shank, projected into the foot transverse plane. This differs from the toe-out angle, which is measured from the long axis of the foot, relative to the line of progression of the body. The foot is defined by the single vector joining the ankle joint centre to the second toe. The relative alignment of this vector and the long axis of the foot is calculated from a static trial using an additional calibration marker from the heel. The foot vector is established by making two rotations about the orthogonal axis. This measure is equal to the angle between the line joining the heel marker and the toe marker, projected in the plane perpendicular to the ankle flexion axis (sagittal). The second rotation is about a foot rotation axis that is perpendicular to the foot vector and the ankle flexion axis. This measure is equal to the angle projected in the plane perpendicular to the foot rotation axis (transverse). The angle is measured between the line joining the heel and toe markers and the line joining the ankle centre and toe marker as previously described [12,19] and according to the protocol stipulated by the Vicon technology in the gait laboratory [20]. Positive values correspond with internal rotation (*Vicon Clinical Manager's User Manual *[20]). Subjects were instructed to walk barefoot at their normal pace over level ground, to capture their natural gait patterns.

### Statistical analysis

Gait data were initially examined for normality and linearity. The peak external knee adduction moment and degree of foot rotation occurring when the adductor moment peaked during early and late stance were averaged over four walking trials. Peak external knee adduction moments were normalised to percentage body weight multiplied by height. Linear regression analyses were used to determine the relationship between meniscal tear presence (yes/no) and severity (grade) (independent variables) and peak external knee adduction moments and foot rotation during early and late stance (outcome variables). Age and gender are associated with meniscal tears and also with gait. Our study used restriction to reduce any confounding associated with gender and included age within our multivariate regression analysis. Moreover, since six participants reported a past knee injury, a history of knee injury (yes/no) was also included in the regression analyses. Furthermore, to see whether rotation effects on the menisci were independent of the adductor moment, this was included within the model. Results in which there were *P *values of less than 0.05 (two-tailed) were considered to be statistically significant. All analyses were performed using SPSS (version 11.0.1; SPSS Inc., Cary, NC, USA).

## Results

Meniscal tears were present in the dominant knees of 9 (45%) of the 20 participating women. Six (30%) of these were located medially and 4 (20%) were located laterally. One woman had a meniscal tear in both medial and lateral compartments. Seven of the 20 women had self-reported a knee injury at some time in their life. No injury occurred in the knee that was imaged. All injuries were reported as mild and did not require any treatment. None of these injuries occurred in the knee imaged. There were no significant differences in the prevalence of meniscal tears (medial *P *= 1.0 and lateral *P *= 0.7), peak external knee adduction moments (early and late stance *P *= 0.8), degree of foot rotation when the adductor moment peaked during early (*P *= 0.4) and late (*P *= 0.7) stance, and age (*P *= 0.14) in women who reported a prior injury and those who did not; however, those with a past injury had slightly lower BMIs (*P *= 0.04). Nineteen of the 20 women had a Kellgren-Lawrence (KL) score of 0 whereas one woman had a KL score of 1. The external knee adduction moment peaked at 12% (early stance) and 48% (late stance) of the gait cycle. Mean gait, meniscal, and subject data are presented in Table 1.

**Table 1:** Demographic and biomechanical mean data

	n = 20
Age, years	60.7 (5.5)
Body mass index, kg/m^2^	25.3 (4.2)
Kellgren-Lawrence grades, number (percentage)	
Grade 0	19 (95%)
Grade 1	1 (5%)
Prevalence of meniscal tears, number (percentage)	9 (45%)
Prevalence of medial meniscal tears, number (percentage)	6 (30%)
Prevalence of lateral meniscal tears, number (percentage)	4 (20%)
Knee adduction moment^a^	
Early stance	4.0 (0.9)
Late stance	2.2 (0.7)
Foot rotation, degrees^b^	
Early stance	-7.65 (6.0)
Late stance	0.44 (5.6)

Values are presented as mean (standard deviation) unless otherwise stated. ^a^Adduction moments are normalised to percentage body weight multiplied by height. ^b^Positive values for foot rotation indicate internal rotation and negative values indicate external rotation.

The peak external knee adduction moment during early stance was positively associated with the presence (*P *= 0.04, *r*^2 ^= 0.3) and severity (*P *= 0.01, *r*^2 ^= 0.4) of medial meniscal tears (Table 2). A trend toward significance was also apparent between the presence of medial meniscal tears and the peak external knee adduction moment during late stance (*P *= 0.09) (Table 2). No association between the presence and grade of lateral meniscal tears during either early or late stance and the peak external knee adduction moment was observed. As 7 of the 20 women had a self-reported knee injury in the past, a history of knee injury was included in the model but did not change the association between meniscal tears and the external knee adduction moment (data not shown).

**Table 2:** Association between external peak knee adduction moment during early and late stance and the presence and severity of meniscal tears

	Univariate regression coefficient (95% CI)	*P *value	Multivariate regression coefficient (95% CI)^a^	*P *Value
**Early stance**				
Any medial meniscal tear y/n^b^	0.8 (-0.1, 1.8)	0.07	1.0 (0.05. 1.9)	0.04
Medial meniscal tear score^c^	0.6 (0.1, 1.1)	0.02	0.6 (0.2, 1.1)	0.01
				
Any lateral meniscal tear y/n^b^	0.3 (-1.5, 0.8)	0.5	-0.3 (-1.5, 0.9)	0.6
Lateral meniscal tear score^c^	-0.1 (-1.0, 0.7)	0.8	-0.1 (-1.0, 0.7)	0.7
				
**Late stance**				
Any medial meniscal tear y/n^b^	0.6 (-0.1, 1.3)	0.09	0.6 (-0.1, 1.4)	0.09
Medial meniscal tear score^c^	0.3 (-0.1, 0.7)	0.13	0.3 (-0.1, 0.7)	0.14
				
Any lateral meniscal tear y/n^b^	0.2 (-1.1, 0.6)	0.6	-0.2 (-1.1, 0.7)	0.62
Lateral meniscal tear score^c^	-0.07 (-0.7, 0.6)	0.8	-0.07 (-0.7, 0.6)	0.8

^a^Adjusted for age. ^b^Increase in peak adduction moment if a meniscal tear is present (tear = 1, no tear = 0). ^c^Increase in peak adduction moment for each increase in grade of meniscal tear score. Adduction moments are normalised to percentage body weight multiplied by height. CI, confidence interval.

No association between meniscal tears and the degree of foot rotation when the external knee adduction moment peaked during early stance was observed (Table 3). However, the degree of foot rotation when the external knee adduction moment peaked during late stance was positively associated with both the presence (*P *= 0.03, *r*^2 ^= 0.3) and severity (*P *= 0.03, *r*^2 ^= 0.3) of medial meniscal tears. The presence of a medial compartment meniscal tear was associated with a 6.2° (95% confidence interval [CI] 0.5 to 11.8; *P *= 0.03) increase in internal foot rotation, and each grade increase in meniscal tear severity was associated with a 3.5° (95% CI 0.35 to 6.6; *P *= 0.03) increase in internal foot rotation (Table 3). When the corresponding peak external knee adduction moment was included in the model, a trend between greater internal foot rotation during late stance and the presence (5.4°, 95% CI -1 to 11.8; *P *= 0.09) and severity (3.0°, 95% CI -0.42 to 6.5; *P *= 0.08) of medial meniscal tears persisted. Moreover, the inclusion of self-report of past history of knee injury in the model did not significantly affect the association between meniscal tears and foot rotation (data not shown).

**Table 3:** Association between foot rotation during early and late stance and the presence and severity of meniscal tears

	Univariate regression coefficient (95% CI)	*P *value	Multivariate regression coefficient (95% CI)^a^	*P *Value
**Early stance**				
Any medial meniscal tear y/n^b^	1.7 (-5.1, 8.5)	0.6	0.16 (-5.6, 8.9)	0.6
Medial meniscal tear score^c^	1.1 (-2.7, 4.9)	0.5	1.1 (-3.0, 5.1)	0.6
				
Any lateral meniscal tear y/n^b^	1.9 (-5.8, 9.6)	0.6	1.9 (-6.2, 9.9)	0.6
Lateral meniscal tear score^c^	1.1 (-4.6, 6.9)	0.7	1.1 (-4.8, 7.1)	0.7
		0.6		
				
**Late stance**				
Any medial meniscal tear y/n^b^	6.3 (1.1, 11.6)	0.02	6.2 (0.5, 11.8)	0.03
Medial meniscal tear score^c^	3.6 (0.6, 6.6)	0.02	3.5 (0.35, 6.6)	0.03
				
Any lateral meniscal tear y/n^b^	2.3 (-4.6, 9.3)	0.5	2.2 (-4.9, 9.3)	0.52
Lateral meniscal tear score^c^	1.0 (-4.2, 6.3)	0.7	1.1 (-4.2, 6.5)	0.6

^a^Adjusted for age. ^b^Increase in early stance peak adduction moment if a meniscal tear is present (tear = 1, no tear = 0). ^c^Increase in peak adduction moment for each increase in grade of meniscal tear score. Positive foot rotation values indicate internal rotation and negative values represent external rotation. CI, confidence interval.

## Discussion

In this cross-sectional study examining women with no clinical knee OA, we have demonstrated that medial meniscal tears are associated with changes in biomechanical factors acting on the medial tibiofemoral compartment during level walking. In particular, the presence and severity of medial meniscal tears were associated with an increased peak external knee adduction moment during early stance and trended toward an association during late stance. Moreover, the presence of medial meniscal lesions was positively associated with the degree of internal foot rotation when the external knee adduction moment peaked during late stance, independent of the magnitude of the adductor moment.

To our knowledge this is the first study to describe a relationship between gait parameters and meniscal tears. We have demonstrated that the presence and severity of medial meniscal tears were positively associated with the peak external knee adduction moment during early stance and trended toward a similar association during late stance. The peak external knee adduction moment is a major determinant of 70% of the total knee joint load passing through the medial tibiofemoral compartment during walking [10], and it is not surprising to have observed these compartment-specific results. Other studies have also demonstrated compartment-specific associations between the peak external adduction moment and other knee joint structures such as the medial tibial plateau area in non-osteoarthritic women [8] as well as medial joint space narrowing in OA populations [11,21] and increased medial compartment cartilage breakdown in rabbits [22].

The presence of medial meniscal tears was also positively associated with the degree of internal foot rotation when the external knee adduction moment peaked during late stance, independent of the magnitude of the adductor moment. We have previously shown that the degree of foot rotation correlates with the knee adduction moment, whereby the magnitude of the peak knee adduction moment during late stance can be reduced by external rotation of the foot [12]. Others have also shown that the magnitude of the toe-out angle (a postural description rather than an isolated joint movement) is inversely associated with the peak external knee adduction moment during late stance [13,14,23]. Therefore, the degree of internal foot rotation during late stance observed in our study may have contributed toward increased medial tibiofemoral joint load by mediating an increase in the peak external knee adduction moment. However, our results demonstrated an association between internal foot rotation and the presence and severity of medial meniscal tears, independent of the peak external knee adduction moment. This suggests that, as well as compressive loads imparted by the knee adduction moment, non-compressive forces such as rotations appear to be an independent determinant of the presence and severity of medial meniscal tears.

This study has demonstrated that gait parameters that isolate medial tibiofemoral joint loads are associated with medial meniscal pathology. It may be that meniscal lesions predict aberrations in gait or alternatively that the gait parameters contributed to the development of these lesions. If the latter were true, our results would imply that by reducing internal foot rotation during late stance, either independent of the knee adductor moment or alternatively by mediating a reduction in the peak external knee adduction moment, meniscal tear prevalence and severity could be reduced. Since meniscal tears are associated with structural changes of OA (including cartilage defect scores, reduced tibial cartilage volume, and increased tibial bone area [2-7,24]), it is possible that modifying the gait parameters examined in this study (for example, via gait retraining or orthoses) may also help to reduce the incidence and burden of knee OA.

The sample size in this study was modest and the range of the 95% CIs was wide, thereby providing the range of uncertainty in our results, however we did have sufficient power to detect a relationship between biomechanical parameters and the presence and severity of meniscal tears. The potential effect of outliers was also examined and shown not to influence the results, and in many cases the 95% CIs also indicate that the true differences could be quite large (if the upper end of the CI is examined). Furthermore, by selecting only healthy middle-aged women, we were able to reduce the effect of potential confounders such as age and gender. The results of this study, however, are limited to non-osteoarthritic women and therefore are not generalizable to men or osteoarthritic populations. Another potential limitation of this study relates to the biomechanical model we adopted. The axis system that measured the magnitude of knee adduction moment and the degree of foot rotation was calculated from the orientation of the shank. Therefore, the knee adduction moment and foot rotation may not have represented independent variables. However, we previously used this model and showed that the relationship between the peak external knee adduction moment and degree of foot rotation is not consistent across stance [12]. We examined a number of associations within this study, but we did not correct for multiple comparisons as this would have severely reduced our power to detect any effects. While it is possible that the significant findings we observed are a result of chance, this is unlikely as the association between meniscal tears and gait remained consistent regardless of which definition of tear we used. In addition, the significant results observed were biologically plausible. Due to our sample size, the relationship between gait, meniscal tears, and any other potential structural changes in the knee was not explored in this study. Larger longitudinal studies examining this would be required as these relationships may not be simply a matter of confounding but rather structural changes on the causal pathway of biomechanic gait abnormalities and knee disease. In addition, it possible that the associations observed are a result of knee injury rather than altered gait; however, while almost one third of our population reported an injury in their knee at some point during their life, all injuries were reported as mild and did not require any treatment. Anyone with severe injuries or symptoms was excluded. In addition, in women who reported any injury to their knee during their life, their contralateral knee was imaged. To confidently determine that a self-report of knee injury was not confounding our results, a history of knee injury was included within the models and did not alter the results, thus implying that the association between adduction moment, foot rotation, and meniscal tear are independent of knee injury. Finally, because of the cross-sectional nature of this study, we are unable to determine cause and effect and therefore cannot conclude whether gait variables caused meniscal lesions or *vice versa*. Longitudinal studies will be required to determine this.

## Conclusion

This study demonstrated a significant positive relationship between the presence and severity of medial meniscal lesions and the magnitude of the peak external knee adduction moment as well as the degree of internal foot rotation during level walking among middle-aged women with no clinical knee OA. Taken together, these results indicate that the presence of medial meniscal tears is associated with changes in biomechanical factors acting on the medial tibiofemoral compartment.

## Abbreviations

ASIS = anterior superior iliac spine; BMI = body mass index; CI = confidence interval; KAD = knee alignment device; KL = Kellgren-Lawrence; MRI = magnetic resonance imaging; OA = osteoarthritis.

## Competing interests

The authors declare that they have no competing interests.

## Authors' contributions

FC and AW were involved in the design and implementation of the study, including data collection and measurement, and were involved in the analysis and interpretation of data. SD, JM-P, J-PP, GJ, and CD were involved in the design and implementation of the study, including data collection and measurement. MD-T and AT were involved in the analysis and interpretation of data. All authors were involved in the manuscript preparation and read and approved the final manuscript.
